# Environmental factors in early childhood are associated with multiple sclerosis: a case-control study

**DOI:** 10.1186/1471-2377-11-123

**Published:** 2011-10-06

**Authors:** Silja Conradi, Uwe Malzahn, Franziska Schröter, Friedemann Paul, Sabine Quill, Eike Spruth, Lutz Harms, Florian Then Bergh, Anna Ditzenbach, Thomas Georgi, Peter Heuschmann, Berit Rosche

**Affiliations:** 1 Department of Neurology & Experimental Neurology, Charité - Universitätsmedizin Berlin, Charitéplatz 1, Berlin, 10117, Germany; 2 Department of Neurology, University Hospital Leipzig, Liebigstraße 20, Leipzig, 04103, Germany; 3 Center for Stroke Research Berlin, Charité - Universitätsmedizin Berlin, Charitéplatz 1, Berlin, 10117, Germany; 4 NeuroCure Clinical Research Center (NCRC), Charité - Universitätsmedizin Berlin, Charitéplatz 1, Berlin, 10117, Germany; 5 Department of Neuropsychiatry and Laboratory of Molecular Psychiatry, Charité -Universitätsmedizin Berlin, Charitéplatz 1, Berlin, 10117, Germany; 6 General Practitioner, Berliner Straße 14b, Berlin, 14169, Germany; 7 General Practitioner, Prenzlauer Allee 90, Berlin, 10409, Germany

## Abstract

**Background:**

Multiple sclerosis (MS) is a chronic inflammatory demyelinating disease of the central nervous system (CNS) with increasing incidence mainly in high-income countries. One explanation of this phenomenon may be a higher prevalence of allergic and autoimmune diseases in industrialized countries as a consequence of otherwise beneficial advances in sanitation (hygiene hypothesis). We investigated environmental factors in early childhood associated with MS.

**Methods:**

A case-control study was performed of 245 MS patients and 296 population-based controls in Berlin. The study participants completed a standardized questionnaire on environmental factors in childhood and youth, including aspects of personal and community hygiene. Multivariable logistic regression analysis was performed to investigate factors in childhood and youth associated with the occurrence of MS.

**Results:**

Mean age was 46 years (range, 20-80) in the MS group and 42 years (range 18-80) in the control group, of which 73.9% in the MS and 61.5% in the control group were female. The multivariable analysis showed that having at least two older siblings (OR 0.54; p = 0.05, for individuals with two older siblings compared to individuals without older siblings), attending a day-care center (OR 0.5; p = 0.004) and growing up in an urban center with more than 100, 000 inhabitants (OR 0.43; p = 0.009) were factors independently associated with a lower chance for MS.

**Conclusions:**

The hygiene hypothesis may play a role in the occurrence of MS and could explain disease distribution and increasing incidence.

## Background

Multiple sclerosis (MS) is a chronic inflammatory demyelinating disease of the central nervous system (CNS), whose incidence has increased over recent decades in some parts of Europe [1-3] and features a specific geographical distribution with higher age standardized incidence rates in northern compared to southern countries [4,5]. Although the etiology remains unclear, MS is widely assumed to be an autoimmune disease in which both genetic susceptibility and environmental factors play a role. For example, in terms of genetics, several studies have pinpointed specific HLA alleles as MS risk genes [6-8]. Environmental factors commonly discussed as potential triggers of the onset or exacerbation of MS include Epstein-Barr virus (EBV), Vitamin D deficiency and smoking [9-13]. Combined, these factors can to some extent explain the north-south gradient of MS incidence [14].

In 1966, Leibowitz et al first suggested that MS might be associated with high sanitation standards during childhood [15], however, subsequent studies on the so-called hygiene hypothesis [16], which proposes that infections in childhood are protective against autoimmune diseases, yielded conflicting results. Fleming et al described an almost inverse geographical relationship between the global prevalence of MS and Trichuris trichiura infection [17]. This human helminth, which is common around the world, can be regarded as a surrogate marker for infections with other macroparasites and for low levels of community sanitation. Although some epidemiological studies related MS incidence to birth order and asthma [18,19], other studies found birth order to have no effect on MS risk in most families and having older siblings as not protective against MS [20].

In light of these conflicting data, we designed our study to broadly investigate the possible association between the incidence of MS and environmental factors in early childhood by assessing key aspects proposed to date in a single, large case-control study.

## Methods

### Participants

In this case-control study, the participating cases had all either had a clinically isolated syndrome or met McDonald's 2005 criteria for MS. They were recruited from the MS out-patient clinic in the Department of Neurology, University Hospital Charité, Berlin, with its two locations in Berlin Mitte and Berlin Steglitz, between 2006 and 2009. We included patients with clinically isolated syndrome, relapsing-remitting, secondary progressive and primary progressive MS at different stages of disease. In total, 429 MS patients were contacted in writing, of which 245 returned signed informed consent forms and completed standardized questionnaires. 33 letters were returned as "address unknown". Thus, the participation rate equaled 62%. Controls were selected from two general practitioner's (GP) practices in the Berlin suburbs of Prenzlauer Berg and Zehlendorf, which cover approximately the same catchment areas as those of the Charité's MS outpatient clinics. The standardized questionnaires, which were identical to those sent to MS patients, were directly distributed to non-MS patients during GP visits by medical receptionists between October and December 2009 (Prenzlauer Berg) and between March and May 2010 (Zehlendorf). While simplifying the logistics of the study, this approach made estimating a response rate impossible. In total, 296 controls were recruited anonymously. The study was approved by the local ethics committee of the Charité - Universitätsmedizin Berlin and all MS patients provided informed consent.

### Questionnaire

The standardized questionnaire completed by MS patients and controls addressed the study participants' medical history regarding asthma, type-1-diabetes and rheumatic diseases. Further questions concerned environmental variables in childhood proposed in the hygiene hypothesis as potential causes of infections. Additionally, the following information was obtained: (a) sex, year of birth, current place of residence, (b) place of birth, place of residence between the ages of 0-6 and > 6-16 years, (c) contact with pets or farm animals between the ages of 0-2, > 2-6 and > 6-16, (d) number and birth order of siblings, (e) day-care attendance during early childhood. The main living environment in youth and childhood was categorized as 'urban' (large towns or cities with *>*100, 000 inhabitants), 'suburban' (towns with 10, 000-100, 000 inhabitants), or 'rural' (villages with *<*10, 000 inhabitants) according to previous studies on allergy diseases [21,22]. Answers were provided from memory and some returned questionnaires were incomplete.

### Statistical analysis

The average age of the MS respondents was slightly higher than the overall average (46 ± 10 years, MS patients vs. 42 ± 10 years, all patients). Proportionally more females than males answered (73.9%, female MS respondents vs. 71.7%, all female MS patients contacted; 26.1%, male MS respondents vs. 29.3%, all male MS patients contacted). There was no difference in response rate between MS patients living in Berlin and those in suburban areas (78%, respondents living in Berlin vs. 78.9%, all patients contacted living in Berlin). The frequency of responses was similar between patients and controls and varied between 90.9% and 99.2% for questions regarding place of residence, siblings, day-care center attendance and contact with pets or farm animals. Unanswered questions were not included in the analysis.

Data analysis was performed using SPSS PASW 18 for Windows. Adjusted odds ratios were estimated using multivariable logistic regression analysis by applying multiple sclerosis as the dependent variable. To include all relevant factors as far as possible and nevertheless comply with the principle of parsimonious modeling, we used an automatic variable selection for model choice. Models were fitted with a stepwise backward selection procedure to avoid an omitted variable error. The goodness of fit of each model was evaluated using the Hosmer and Lemeshow test. All p-values were two-sided. Because multiple sclerosis is a very rare disease (and therefore the odds ratio is a good approximation of the relative risk), we also calculated the sequential and average attributable fractions (ASAF) for each factor detected as significant influencing factor in our multivariable logistic regression analysis. Using ASAF, attributable fractions can be calculated in a multivariable setting independently of the order in which the individual factors are included in the model [23,24].

## Results

Questionnaires from 245 MS patients and 296 controls were evaluated. The average age of the MS respondents was slightly higher than the overall average (46 ± 10 years, MS patients vs. 42 ± 10 years, all patients). Proportionally more females than males answered (73.9%, female MS respondents vs. 71.7%, all female MS patients contacted; 26.1%, male MS respondents vs. 29.3%, all male MS patients contacted). There was no difference in response rate between MS patients living in Berlin and those in suburban areas (78%, respondents living in Berlin vs. 78.9%, all patients contacted living in Berlin). Descriptive characteristics of cases and controls are shown in Table 1. Age at diagnosis and gender proportions of the MS patients were typical for the disease and fit with previous studies (data not shown) [25,26]. Our controls showed a higher proportion of men in comparison to the group of MS patients. Statistically we adjusted for this by including the variable gender in our multivariable analyses.

**Table 1:** Demographic data of all patients and controls

Characteristic	Controls, N = 296	MS patients, N = 245
	n (%)	n (%)
Age, years		
Median (IQR)	40.0 (27-54)	46.0 (37-54)
Age group, (based on sample quartiles)		
≤ 31	107 (36.3)	29 (11.8)
32-43	63 (21.4)	73 (29.8)
44-54	52 (17.6)	84 (34.3)
≥ 55	73 (24.7)	59 (24.1)
Female	182 (61.5)	181 (73.9)
Day care 0-3	117 (40.3)	61 (25.4)
Past medical history		
Asthma	24 (8.1)	6 (2.4)
Diabetes mellitus	4 (1.4)	5 (2.0)
Rheumatic diseases	6 (2.0)	3 (1.2)
Residence age 0-6		
< 10000	24 (8.9)	42 (17.7)
10000-100000	68 (25.3)	61 (25.7)
> 100000	177 (65.8)	134 (56.5)
Number of older siblings		
0	135 (46.7)	125 (51.7)
1	76 (26.3)	82 (33.9)
2	49 (17.0)	31 (12.8)
≥ 3	29 (10.0)	4 (1.7)

All results of the univariable and multivariable analyses are shown in Table 2. Univariable analysis showed our control group had a significantly higher proportion of participants with a history of asthma compared with our study's MS patients (OR 0.29; 95% CI 0.11-0.71; P = 0.004 for univariable analysis). However, because the hygiene hypothesis does not specify asthma as a risk factor for MS we chose to exclude it from further analysis.

**Table 2:** Results of univariable and multivariable analysis for different risk factors

	Univariable			Multivariable^§^		
	**OR**	**95% CI**	**P-value^#^**	**OR**	**95% CI**	**P-value**

**Age**			< 0.0005			< 0.0005
**≤ 31**	1.00			1.00		
**32 - 43**	4.28	2.51-7.27	< 0.0005	4.44	2.48-7.94	< 0.0005
**44 - 54**	5.96	3.49-10.19	< 0.0005	6.62	3.59-12.24	< 0.0005
**≥ 55**	2.98	1.75-5.09	< 0.0005	2.88	1.54-5.38	0.001
**Female**	1.77	1.23-2.56	0.002	1.69	1.09-2.61	0.018
**Birth order**			< 0.0005			0.001
**1**	1.00			1.00		
**2**	1.17	0.78-1.73	0.481	0.93	0.59-1.47	0.760
**3**	0.68	0.41-1.14	0.159	0.54	0.30-1.00	0.050
**≥ 4**	0.15	0.05-0.44	< 0.0005	0.08	0.02-0.30	< 0.0005
**Day care age 0-3**	0.50	0.35-0.73	< 0.0005	0.50	0.31-0.80	0.004
**Residence age 0-6**			0.011			0.015
**< 10000**	1.00			1.00		
**10000-100000**	0.51	0.28-0.94	0.034	0.67	0.33-1.35	0.262
**> 100000**	0.43	0.25-0.75	0.003	0.43	0.23-0.81	0.009

The multivariable analysis showed that having at least two older siblings had a protective effect against MS compared to individuals without older siblings (OR 0.54; p = 0.05). This suggests that being born late in sibling birth order protects against MS. Although we found that having at least 3 older siblings had an extremely significant effect, it should be noted that only 4 of the MS patients in our study matched this factor. Similarly, day-care center attendance between the ages of 0-3 years also showed a decreased risk for MS (OR 0.5; p = 0.004).

We found a negative association between living in urban centers with more than 100, 000 inhabitants between the ages of 0-6 years and MS status (OR 0.43, CI 0.23 - 0.81, p = 0.009). This high significance factor has to be interpreted cautiously because 96% of our controls and only 82% of our cases currently live in a city with > 100, 000 inhabitants. Furthermore, 65% of those who currently live in a city with > 100, 000 inhabitants also lived in a city with > 100, 000 inhabitants between the ages of 0-6 years, whereas only 32.7% of those who currently live in a city with < 100, 000 inhabitants lived in a city with > 100, 000 inhabitants during their early years. Overall, however, the data showed a significant MS risk for patients from rural areas, even when this recruitment bias was included in the statistical analysis.

The multivariable logistic regression analysis showed that an age of ≥32 years, being female, being first or second child of a family, not having attended a day-care center and having grown up in a rural area with less than 100, 000 inhabitants can be considered as dichotomous risk factors, for which we calculated attributable risks within our multivariable setting using average sequential attributable fractions. According to our analysis 89.6% of MS cases can be attributed to the factors "age ≥32 years" (36.6%), "female" (11.4%), "no day-care attendance age 0 - 3 years" (11.6%), "residence with < 100.000 inhabitants age 0 - 6 years" (6.7%) and "first or second child in birth order" (23.3%). Furthermore, well over half of the MS cases (59.9%) can be attributed to the two factors "age ≥32 years" and "first or second child in birth order". The results of the attributed risks of the identified risk factors are shown in Table 3.

**Table 3:** Attributable risks of identified risk factors

Factor	Attributable fraction (%)
**Age > 32**	36.6
**Female**	11.4
**No day-care attendance age 0-3**	11.6
**Residence < 100.000 inhabitants age 0-6**	6.7
**Born as first or second child**	23.3

The questionnaire also surveyed the study participants' contact with pets or farm animals during different phases of childhood and youth as a possible source of pathogenic germs. No association could be found between the exposure to pets or farm animals and MS incidence.

## Discussion

This case-control study identified a number of environmental factors in childhood and youth significantly associated with MS occurrence in later life (Table 2). We found a strong inverse relationship between having at least two older siblings and MS status, with a high statistical significance for having at least three older siblings. This could mean that younger siblings have a reduced risk for developing MS because they have early contact with pathogen germs via older siblings. We also found a strongly significant negative association between attending day care between the ages 0-3 years and MS status. This result is similar to data for atopic diseases, which show that first-born children appear to be at increased risk of allergic disease [27]. For MS, one study has shown that high exposure to younger infant siblings was protective for MS [18], although another study was unable to confirm this effect [27-30]. The data underline that the contact with other children has to take place as early as possible, which would explain why older siblings are not in turn protected by their younger siblings.

These data lend weight to the hygiene hypothesis, since older siblings and other children at day care provide multiple sources of infection. They could also be related to the finding that high infant exposure reduces the risk of infectious mononucleosis and elevated EBV IgG levels, given that infectious mononucleosis and elevated EBV IgG levels have been identified as MS risk factors [18,31-33].

We hypothesized that growing up in an urban center is a risk factor for MS, given the data from various urban-rural allergy studies and the studies by the group of Beebe, who found a higher incidence of MS in urban areas [34]. It is generally accepted that potential infections are more frequent in rural compared to urban areas due to more frequent contact with pets or farm animals and less sanitary equipment. However, surprisingly, growing up in areas < 100 000 inhabitants showed an association with an increased MS risk in our study. Because the MS patients were recruited from our MS center in Berlin and the controls were also recruited from two Berlin-based general practitioners in Berlin, study participants that had grown up in an urban center were overrepresented, a fact that was adjusted for in the analysis. Similar observations to ours have been made in France, where Fromont et al found a lower prevalence of MS in Paris than in its surroundings [35]. The latter study proposed the high proportion of immigrants in Paris as an explanation for the finding, but admitted that this did not explain the north-south geographical distribution. We believe that it might be a mistake to take the rural-urban comparison as a proxy for the hygiene hypothesis. In developed countries such as Germany, the sanitary standards and probability of infections does not differ between residents of rural areas compared to those in urban centers. On the contrary, more contact with children of the same age and public transport, the risk of infection with pathogens may be higher in big cities. However, further investigations of relevant infectious agents are necessary to answer this question decisively.

Our data show that asthma appears to be a protective factor in MS and confirm other studies that found a reduced prevalence of asthma in MS patients [26]. This observation fits with the concept that asthma is Th2-related and MS is Th1-related [36]. The prevalence of asthma is higher in urban areas, due to air pollution and climate change [37,38]. Whether asthma results in protective immunological changes against Th1-related diseases like MS or whether instead MS is protective against asthma has not yet been elucidated. Both diseases are multifactor-induced and complex immunological interactions cause an autoimmune reaction that the Th1-Th2 concept cannot fully explain.

As our study included a wide range of MS patients treated in our outpatient clinics, with different variations of the disease and disease duration, we could not determine whether the environmental factors pertain only to specific subgroups of patients or have an influence on disease onset. Because only 62% of MS patients answered the questionnaire and the response rate was higher for older patients, a selection bias may be present. Including only patients with a recent MS diagnosis would be a means of preventing this bias but would complicate recruiting a sufficient number of patients. Two further possible limitations could have resulted from, firstly, the difference between the recruitment strategies for MS patients and controls and secondly, from the fact that, as the control subjects were recruited from patients visiting general practitioners, they may exhibit a higher prevalence of other autoimmune diseases than healthy controls. It should be noted that further environmental risks factors, which were not included in our questionnaire, have also been discussed for MS. To accurately gauge the relevance of individual factors to overall MS risk, all factors should be considered in future studies. Finally, although the distribution of patients and controls is similar, the missing matching is a further limitation of our study. Larger case-control studies in the future could answer this question.

## Conclusion

This study indicates "day-care attendance at age 0-3 years", "residence in an urban center with > 100.000 inhabitants at age 0-6 years" and "at least third child in birth order" are protective factors in relation to MS and supports the hygiene hypothesis. These findings are in line with results for other autoimmune diseases and suggest that this direction of investigation towards understanding the increasing incidence of autoimmune disorders is indeed highly promising.

## Abbreviations

MS: multiple sclerosis; CNS: central nervous system; OR: odds ratios.

## Study sponsorship or funding (industry and institutional)

The pilot study was conducted at Charité - Universitätsmedizin Berlin, Germany.

## Competing interests

SC has no competing interests. UM has no competing interests. FS has no competing interests. FP has received research support and speaker honoraria from Teva/Sanofi Aventis, Bayer Schering Pharma and Merck Serono. His work was supported by the German Research Foundation (DFG Exc 257 to FP). SQ attended meetings sponsored by Biogen Idec, Bayer and Teva Pharmaceuticals. ES has no competing interests. LH has received lecture honoraria from Biogen Idec, MerckSerono and Teva Pharmaceuticals. FTB has received research support from Teva Neuroscience. AD has no competing interests. TG has no competing interests. PH has no competing interests. BR participated in meetings sponsored by and received lecture honoraria from Biogen Idec, Bayer, Merck Sorono and Teva Pharmaceuticals and receives research support from Bayer Schering Pharma.

## Authors' contributions

CS contributed to writing the manuscript, the study concept and design and the analysis and interpretation of data. UM contributed to writing the manuscript and the analysis and interpretation of data. FS contributed to the study concept and design and the analysis and interpretation of data. FP, SQ, ES, LH, FTB, AD and TG contributed to writing the manuscript. PH contributed to writing the manuscript, the study concept and design and the analysis and interpretation of data. BR contributed to writing the manuscript, the study concept and design and the analysis and interpretation of data. All authors read and approved the final manuscript.

## Pre-publication history

The pre-publication history for this paper can be accessed here:

http://www.biomedcentral.com/1471-2377/11/123/prepub
